# Editorial: Ionotropic Glutamate Receptors Trafficking in Health and Disease

**DOI:** 10.3389/fncel.2016.00242

**Published:** 2016-10-28

**Authors:** Milos Petrovic, Inmaculada M. Gonzalez-Gonzalez, Jeremy M. Henley

**Affiliations:** ^1^Institute of Medical Physiology, School of Medicine, University of BelgradeBelgrade, Serbia; ^2^Centro de Investigacion Medica AplicadaPamplona, Spain; ^3^Centre for Synaptic Plasticity, School of Biochemistry, University of BristolBristol, UK

**Keywords:** ionotropic glutamate receptors, trafficking, NMDA, AMPA, kainate

Because of their fundamental role in excitatory synaptic function in health and disease, ionotropic glutamate receptors (iGluRs) continue to be the focus of wide-spread research efforts within the neuroscience community.

A core aspect of on-going research is the elucidation of the complex sequence of events that coordinate iGluR processing, delivery to, retention at, recycling, and removal from synapses (collectively known as receptor trafficking). Understanding of the activity dependent regulation of these events in healthy and diseased neurons will likely provide new targets for therapeutic intervention and, we believe, holds tremendous promise for new and improved treatments for neurological and neurodegenerative diseases.

The contributors to this special issue “Ionotropic glutamate receptors trafficking in health and disease” each provide new insights into different aspects of this complex problem, covering a wide range of issues, starting with early stages of trafficking taking place in the ER, through the distribution of receptors along actin tracks to the final stages of insertion into the surface membrane. Taken together with broader overviews, these papers provide a broad picture of current understanding of how postsynaptic iGluRs are integral to the initiation and expression of synaptic plasticity and how this impacts on disease.

With respect to NMDAR subunits, specific sections have been shown to affect the transition between individual steps in receptor trafficking, including their processing and eventual release from the ER. Based on their structure, different rules may apply to individual subunits and it is now shown that specific structural features of GluN2C can also regulate this process (Lichnerova et al.). On a wider scale, the processes surrounding the ER-related events in NMDAR trafficking are also covered by a dedicated article in our special issue (Horak et al.).

Beyond their regulation at the level of transcription and translation, iGluRs are subject to stringent regulation by post-translational modifications. Among these, the phosphorylation of GluN2A at Ser1048 by the Dual specificity tyrosine-phosphorylation-regulated kinase 1A (DYRK1A) can interfere with the internalization of GluN1/GluN2A NMDARs, while also potentiating their activation and increasing the NMDAR-current density (Grau et al.).

The complexity of excitatory synapses is enhanced by AMPAR- and NMDAR-interacting proteins. These interactions can be prolonged or transient and have profound effects on trafficking. For example, carnitine palmitoyltransferase 1C (CPT1C) affects early steps of AMPAR trafficking to control AMPAR availability at synapses (Gratacòs-Batlle et al.).

Another intriguing phase of AMPAR trafficking is their forward transport along tracks provided by rapidly changing actin cytoskeleton. This occurs with the participation of various interacting proteins including PICK1 and the ARP2/3 Complex, ADF/Cofilin, as well as molecular motors, such as myosin. All of these interactors have the ability to modify trafficking of AMPARs, thus determining both the basal synaptic transmission, as well as activity-dependent regulation of synaptic strength (Hanley). Once that the receptors reach their targeted surface membrane, they need to be inserted in it and this regulated process, mediated by SNARE proteins, is critical for the postsynaptic expression of various forms of plasticity, as reviewed by Jurado and Chater and Goda.

An emerging concept is that iGluRs interact with other neurotransmitter systems and their receptors, including GABAB (Kantamneni), nicotinic receptors (Zappettini et al.), as well as dopaminergic system. This crosstalk has far-reaching implications, especially for diseases such as Parkinson's and Huntington's, as well as in addiction (Gardoni and Bellone).

Needless to say, with so many roles in synaptic function, it is clear that disturbances in iGluR trafficking give rise to serious neurological and psychiatric diseases. Among proteins affecting iGluR trafficking, SynGAP (and the genetic changes affecting its expression) is now recognized as the pathophysiological substrate for autism spectrum disorder and this fascinating topic is reviewed within our issue (Jeyabalan and Clement).

In conclusion, we believe that the articles presented in this special issue represent a valuable resource that provides a clear overview of the current state of the art of this important and rapidly progressing field of neuroscience.

## Author contributions

All authors listed, have made substantial, direct and intellectual contribution to the work, and approved it for publication.

### Conflict of interest statement

The authors declare that the research was conducted in the absence of any commercial or financial relationships that could be construed as a potential conflict of interest.

